# On the way of revealing coactivator complexes cross-talk during transcriptional activation

**DOI:** 10.1186/s13578-016-0081-y

**Published:** 2016-02-24

**Authors:** Aleksey N. Krasnov, Marina Yu. Mazina, Julia V. Nikolenko, Nadezhda E. Vorobyeva

**Affiliations:** Department of Transcription Regulation and Chromatin Dynamic, Institute of Gene Biology, Russian Academy of Sciences, Moscow, 119334 Russia

**Keywords:** Transcription, RNA polymerase II, Coactivator complexes, Preinitiation complex, Chromatin, Remodeling

## Abstract

Transcriptional activation is a complex, multistage process implemented by hundreds of proteins. Many transcriptional proteins are organized into coactivator complexes, which participate in transcription regulation at numerous genes and are a driver of this process. The molecular action mechanisms of coactivator complexes remain largely understudied. Relevant publications usually deal with the involvement of these complexes in the entire process of transcription, and only a few studies are aimed to elucidate their functions at its particular stages. This review summarizes available information on the participation of key coactivator complexes in transcriptional activation. The timing of coactivator complex binding/removal has been used for restructuring previously described information about the transcriptional process. Several major stages of transcriptional activation have been distinguished based on the presence of covalent histone modifications and general transcriptional factors, and the recruitment and/or removal phases have been determined for each coactivator included in analysis. Recruitment of Mediator, SWItch/Sucrose Non-Fermentable and NUcleosome Remodeling Factor complexes during transcription activation has been investigated thoroughly; CHD and INOsitol auxotrophy 80 families are less well studied. In most cases, the molecular mechanisms responsible for the removal of certain coactivator complexes after the termination of their functions at the promoters are still not understood. On the basis of the summarized information, we propose a scheme that illustrates the involvement of coactivator complexes in different stages of the transcription activation process. This scheme may help to gain a deeper insight into the molecular mechanism of functioning of coactivator complexes, find novel participants of the process, and reveal novel structural or functional connections between different coactivators.

## Background

Transcriptional machinery includes hundreds of transcription factors that function coordinately to provide for the progression of the multistage transcriptional process. To date, ample data have been accumulated on the functional properties of multiprotein transcription coactivator complexes that cannot themselves specifically interact with DNA but are nevertheless indispensable for transcriptional activation [1, 2]. Unfortunately, most studies on these complexes are focused on their effect on transcription in general. Initial characterization of a coactivator protein or complex is usually limited to experiments on whether it is necessary for activating and maintaining the transcription activation of a certain gene type, while data on the particular stage of the transcriptional process at which this coactivator functions are scarce or absent. Hence, a specific opinion about the process of transcriptional activation has been formed. It is currently considered that transcriptional activation is initiated by a limited set of coactivator complexes, while different research groups have shown that this process is cyclic and that coactivator complexes do not bind to promoters for a long time but replace each other in its course [3]. Such a mobile action pattern suggests that the number of protein complexes involved in transcriptional activation is probably greater than considered previously. Kinetic methods applied to the study of these complexes may help to improve and expand current knowledge of transcriptional activation.

This review is focused on the data concerning the involvement of different coactivator complexes at particular stages of transcriptional activation, which have been obtained by different authors in experiments on different organisms. Its purpose is to integrate these data into a preliminary model of transcriptional activation, which is not intended to describe this process for any real gene but rather is an attempt to generalize all the relevant information available from different sources. Hopefully, the proposed model will help to find new directions for research on the functions of coactivators and their participation in the transcription cycle.

## Basic stages of transcriptional activation

**Fig. 1:**
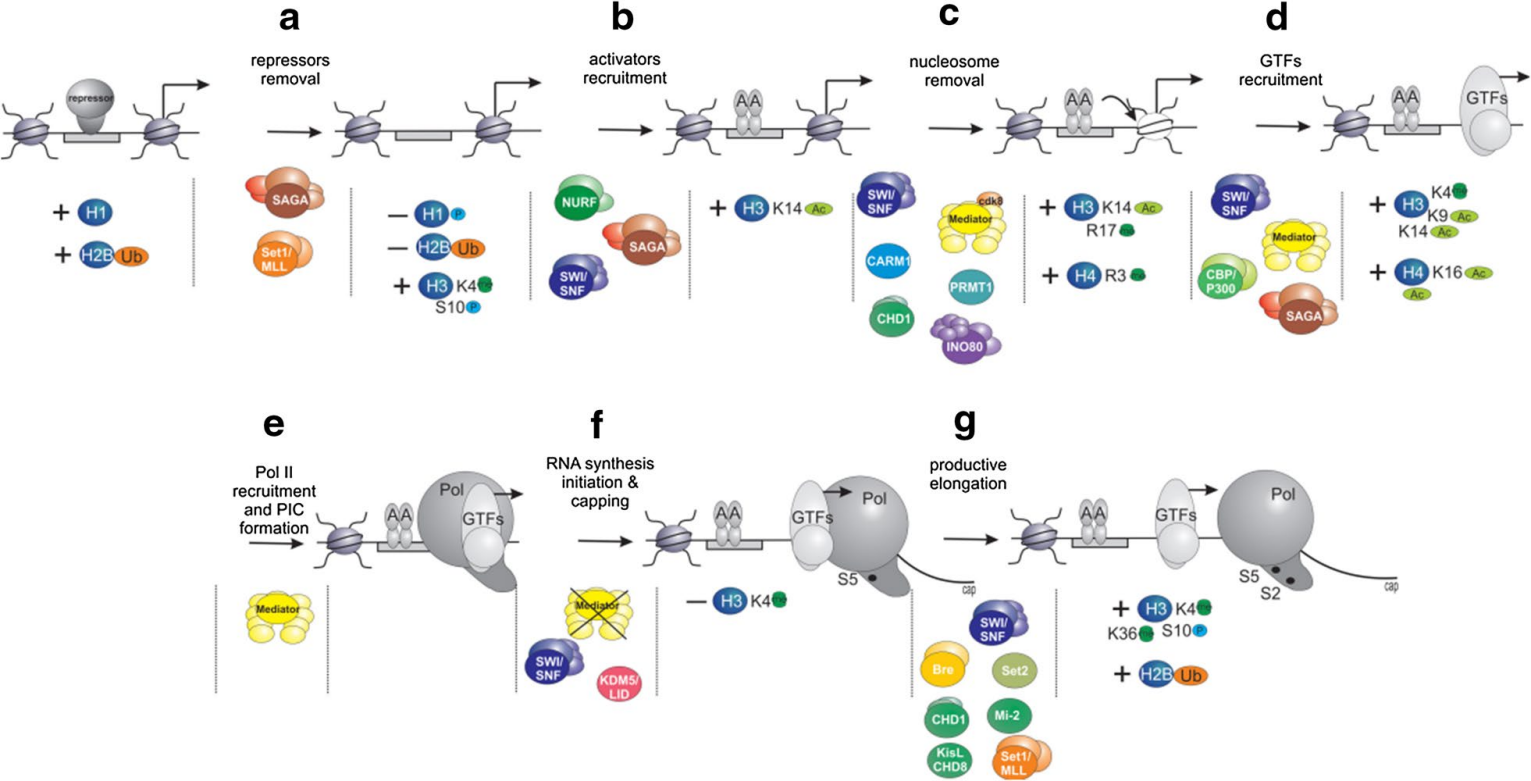
A scheme describing coactivator complexes participation in different stages of transcription activation cycle (scheme is described in detail in the body of the manuscript). In the presented scheme, two groups of symbols show coactivator complexes involved in each step of transcriptional activation and specific chromatin modifications characterizing different states of the gene between these steps. Transcription activation cycle includes following steps: repressors removal **a** activators recruitment **b** nucleosome removal **c** general transcription factors (GTFs) recruitment **d** Pol II recruitment and preinitiation complex (PIC) formation **e**, RNA synthesis initiation and capping **f** and transition into productive elongation **g** Following coactivator complexes are participated in transcription activation process: Mediator, *NURF* nucleosome remodeling factor, *SWI/SNF* switch/sucrose non-fermentable complex, *INO80* INOsitol auxotrophy 80 complex, *CHD1* chromodomain-helicase-DNA-binding protein 1 complex, *Mi-2* complex formed by dermatomyositis-specific autoantigen Mi-2, *KisL/CHD8* complex formed by Kismet/chromodomain-helicase-DNA-binding protein 8. Following chromatin modifications mark different states of the gene between steps of transcription activation: *H1P* phosphorylated histone H1, *H2BK120Ub* ubiquitinylated at K120 histone H2B, *H3K4me3* trimethylated at K4 histone H3, *H3S10P* phosphorylated at Ser10 histone H3, *H3K14Ac* acetylated at K14 histone H3, *H3R17me2*–dimethylated at Arg17 histone H3, *H4R3me2* dimethylated at Arg3 histone H4, *H3K9Ac* acetylated at K9 histone H3 and *H3K36me3* trimethylated at K36 histone H3. These chromatin modifications are introduced/removed by following chromatin modifiers: *SAGA* Spt-Ada-Gcn5 acetyltransferase complex, *Set1/MLL* [Su(var)3-9, enhancer-of-zeste, Trithorax]1/mixed-lineage leukemia complex, *CARM1* coactivator associated arginine methyltransferase 1, *PRMT1* protein arginine methyltransferase 1, *CBP/p300* CREB binding protein, *KDM5/Lid* lysine-specific demethylase 5, *Bre* complex formed by BREfeldin A-sensitivity protein 1, *Set2* [Su(var)3-9, enhancer-of-zeste, trithorax]2 protein

Activation of gene transcription is a multistage process, but for clarity in data presentation, we concentrate here only on its basic steps that are likely to occur during activation of the majority of inducible genes (Fig. 1). In the presented scheme, two groups of symbols show coactivator complexes involved in each step of transcriptional activation and specific chromatin modifications characterizing different states of the gene between these steps.

Transcription activation processes begin with the removal of repressor complexes from the promoter (Fig. 1a). Since there are different ways of transcriptional repression, several types of such complexes may be associated with genes [4]. They are not described in detail here because of space limitations, the more so that the data on repressors and corepressors are voluminous and deserve a review of their own.

At the next step, a DNA-binding transcriptional activator binds the promoter (Fig. 1b). Transcriptional activators are a group of proteins that may differ structurally but share the same functions. They recognize specific DNA sequences and provide for the recruitment of coactivator complexes, ensuring the selectivity of their action and the consequent transcriptional output [5]. The best-studied eukaryotic activators are nuclear receptors, which function in the form of homo- or heterodimers [6].

The transcriptional activator interacts with the promoter to stimulate the subsequent recruitment of chromatin remodeling complexes (a special class of coactivator complexes). Under their influence, the promoter sequence is cleared of nucleosomes (Fig. 1c). This step is indispensable for the progression of transcriptional activation, since the nucleosomes occupying the promoter of the gene prevent preinitiation complex formation and RNA polymerase II (Pol II) binding [7, 8]. The stages of activator recruitment and promoter clearance are united in some models of transcriptional activation [9]. The possibility has been suggested that coactivators are recruited in the form of a preformed complex with the transcriptional activator. According to this mechanism, coactivator complexes are involved in preliminary chromatin remodeling, which facilitates the subsequent interaction between the promoter and activator. Both of the promoter remodeling mechanisms can be realized in vivo for different types of genes.

General transcription factors (GTFs) are recruited at the next step of transcriptional activation, which results in Pol II recruitment and preinitiation complex (PIC) formation. This process has been well studied to date, and its detailed three-dimensional models have been recently proposed by two independent research groups [10, 11]. Recruitment of general transcription factors and formation of the preinitiation complex are regarded as individual stages in the transcriptional activation process (Fig. 1d, e).

Pol II can be stably associated with the promoter as an inactive complex, but its recruitment usually leads to the initiation of transcription (Fig. 1f). This step is associated with a covalent modification of the largest Pol II subunit (phosphorylation of serine 5 in the C-tail domain). When the newly synthesized transcript is about 30 nucleotides long, Pol II leaves the promoter, disrupting all interactions with the preinitiation complex, while the transcript undergoes capping at the 5′ end, which protects it from degradation by exonucleases. Such a state of the gene promoter may be highly stable. The majority of eukaryotic inactive genes remain at this stage (early transcript elongation), which is referred to as Pol II pausing [12, 13]. Apparently, this type of transcriptional regulation is characteristic of genes that need to be activated fast and synchronously (developmental and stress-inducible genes).

At the next step, active transcription elongation takes place (Fig. 1g). The initiation of this process is associated with further modification of the largest subunit of the Pol II complex, namely, phosphorylation of serine 2 in the C-tail domain, with the phosphorylation level increasing toward the 3′ end of the gene sequence. Active transcription elongation is accompanied by specific chromatin modification (particularly H3K36me3 and H2BK120Ub) in the transcribed gene region.

## Nuclear dynamic of the transcriptional complexes

For a long time, studies of protein kinetics in transcription have generally been limited to two methods: FRAP and ChIP (fluorescence recovery after photobleaching and chromatin immunoprecipitation) [14–16]. The first, FRAP, allows for investigations in individual cells with high temporal resolution, whereas the second, ChIP, can only be used with populations of cells but provides the opportunity to investigate several factors in a single experiment. Data obtained with FRAP have demonstrated that most of the described transcriptional proteins are not able to bind DNA for more than a minute [17]. These results are not consistent with the ChIP data in which the transcriptional factor residence times on the chromatin reach tens of minutes and result in the formation of “slow” recruitment cycles [18]. To explain this discrepancy, whole interactions have been divided in two types. Fast-binding interactions observed with the FRAP technique are believed to be unproductive in most cases, whereas longer interactions registered by ChIP are thought to be productive and to be the actual drivers of transcriptional activation [19]. Later, this conception was questioned by results from Karpova and colleagues who demonstrated that fast interactions of activator proteins with DNA can also be productive [20]. Moreover, fast interactions observed with FRAP are actually suspected to be the cause of the observed “slow” cycling of transcription factors [21]. It could be that strong interactions of transcriptional proteins with DNA do not occur at all. The registration of cycling in ChIP experiments has been suggested to result from the high degree of synchronicity of the transcriptional processes in cell populations, which results in the simultaneous progression of the promoters through the same stages. In other words, the chromatin of the promoter at different transcriptional stages could vary in the “competency” of the binding of some transcriptional factors, which would result in the peaks observed with the ChIP technique.

With time, the “slow” recruitment of transcriptional complexes has found an explanation in a contemporary model of the transcriptional process [22]. The patterns and periodicities of transcriptional complexes correlate well with the formation of transcriptional “bursts” in eukaryotic cells [23]. There are models that attempt to simulate the “bursting” process of transcription. Notably, such models describe the possible existence of a “slow” cycle of transcriptional complex recruitment that is similar to which has been observed in ChIP studies [22]. In these models, each transcriptional complex binds the promoter for a relatively short time. However, these interactions leave some long-living “outputs” (for example, some changes in the chromatin structure) that in turn stimulate the recruitment of the subsequent complex. The promoter “competence” for the binding of different transcriptional complexes could be changed during the transcriptional “burst”. Such changes would result in the sequential binding of the corresponding complex and stimulation of one or another stage of the activation cycle. This increase in transcription effectiveness could be the mechanism of “burst” development. Indeed, recent experimental data demonstrate that transcriptional regulation is implemented not by a single rate-limiting step but as a two- or even multi-step process [24, 25]. In other words, transcription regulation can be executed at different phases of the process.

The palette of the methods that can be used to investigate transcriptional protein dynamics has greatly expanded in recent times. Contemporary spectroscopic techniques enable research into the actions of the individual proteins in individual living cells or even in their parts [26, 27]. These methods are generally used to analyze proteins that directly bind DNA, but some coactivator complexes have also been examined [28]. The application of these to the investigation of protein dynamics at individual loci or even genes would help to clarify the dynamics of proteins during the transcriptional process. The crosslinking kinetics (CLK) assay was developed rather recently and enables the investigation of protein binding dynamics at individual genes [29]. The primary results obtained with this novel method have demonstrated that the half-life of the interaction of a transcriptional protein with the chromatin can significantly depend on the gene it is currently bound to. The CLK method could help to obtain missing experimental data that are required to model the participation of proteins in transcription. The ChIP method has progressed recently. Currently, the profiles of protein-DNA binding can be acquired for individual cells [30]. These advances allow for the estimation of transcriptional “noise” that is a difference in the progression of transcription in different cells of a population during ChIP-based investigations [31]. Further development of this technique could lead to significant improvements in its temporal resolution, which would enable the detection of the “fast” cycles. Much work related to the determination of the real dynamic characteristics of transcription awaits researchers. The gene-specificity of this process together with possible differences in the dynamics of different subunits of the same complex make this task rather complex. However, recent breakthroughs, particularly in single-cell methods, nourish the hope for the forthcoming identification of opportunities to investigate the dynamic of proteins during the transcriptional activation process in real-time.

## Chromatin modifications as specific markers of different stages of transcriptional activation

For a long time, scientists investigating the transcription cycle have been searching for additional markers that could help them to distinguish between its different stages. Eventually, covalent chromatin modification has proved to be the most promising marker. More than a decade ago, the correlation between the state of promoter chromatin and gene activity was discovered, and the “histone code” hypothesis was advanced to explain the observed results [32]. According to this hypothesis, the state of chromatin plays an essential role in the regulation of transcription, with histone modifications being responsible for the recruitment of transcriptional proteins and thereby determining the transcriptional status of the gene. However, a number of subsequent experimental studies have shown that chromatin modification is a dynamic process, and it now seems unlikely that histone modifications alone can account for coactivator recruitment. It is likely that, histone modifications support the dynamics of the transcriptional process: coactivators are stimulated by recognizing these modifications and then interact with the promoter in a dynamic way, replacing each other during the transcription cycle. In this review, histone modifications are considered as additional markers of particular stages of gene transcription that occur in a given moment of time.

The correlation between histone modifications and the course of transcription has been evaluated in detail in studies on the transcription activation cycle kinetics, including an analysis of promoter chromatin status at different transcriptional stages. In the pioneering study on the molecular mechanism of pS2 gene activation by estrogen receptor, Metivier and colleagues have shown that several histone modifications (such as histone H4 methylation at Arg3 and histone H3 acetylation at Lys14) are introduced into the promoter chromatin at the beginning of the transcription cycle, immediately after activator recruitment but prior to preinitiation complex formation [18]. Histone H3 methylation at Arg17 and histone H4 acetylation at Lys16 take place after general transcriptional factor recruitment but before RNA polymerase II binding. A termination of the transcription initiation process results in the removal of RNA polymerase II and all histone modifications from the promoter. The observed data on the state of chromatin at different stages of the transcription cycle are summarized by these authors in a special review [33].

Another detailed kinetic research paper is focused on the dynamics of coactivators during progesterone receptor-dependent activation and, in particular, on the primary molecular mechanisms of transcription initiation immediately after the input of activation signal [9]. Its results show that repressive complexes and histone H1, which facilitates the formation of a tight chromatin structure, are removed from the promoter within the first minute after activation. Histone H1 phosphorylation is the first histone modification introduced into the promoter chromatin and appears to be the primary chromatin marker of the activation process [34]. Histone H3 trimethylation at Lys4 also takes place at earliest steps of transcription initiation, before the removal of promoter-bound nucleosomes. A complex consisting of progesterone receptor and NURF remodeling coactivator is recruited after the above modifications, being followed by the SWI/SNF chromatin remodeling complex that clears the promoter of nucleosomes. The preinitiation complex is formed on progesterone-dependent promoters due to the coordinated action of SWI/SNF and NURF coactivators.

Unfortunately, no other detailed studies are available that describe the kinetic of transcriptional activation and summarize chromatin modifications during the transcription process, but there are several pieces of evidence that support the concept that the introduction of histone modifications is correlated with particular stages of transcription.

As noted above, histone H3 trimethylation at Lys4 (H3K4me3) takes place at the earliest stages of progesterone-dependent gene transcription initiation [9]. It has recently been confirmed that this modification is implemented by the COMPASS complex at the beginning of activation, right after the removal of repressive complexes [35]. Moreover, the H3K4me3 modification has proved to be also important for other stages of transcription, namely, preinitiation complex formation and transition to elongation. The interaction of TAF3 (a TFIID subunit) with the H3K4me3-modified histone tail supports tight binding of this general transcriptional factor with promoters [36–38]. This observation is supported by the results of studies on plants showing that H3K4me3 methyltransferase is important for the TFIID recruitment [39]. It has been found, however, that the activity of these enzymes is not necessary for supporting preinitiation complex formation in plant cells. Thus, the association between the H3K4me3 modification and the recruitment of general transcription factors has not been confirmed unequivocally. The significance of the H3K4me3 modification for the latest stage of transcription activation, transition into elongation, has been demonstrated in several organisms, including plants and *Drosophila* [39–41]. Unfortunately, the molecular mechanism accounting for the role of this modification during Pol II complex transition from the initiation to elongation stage has not yet been revealed.

Acetylation was the first covalent posttranslational modification described for the histones [42]. It was initially considered that such a modification itself contributes to transcriptional regulation by neutralizing the positive charge of the histone tails and thereby weakening their interaction with DNA, with consequent chromatin relaxation [43]. As shown in subsequent studies, however, the main point is that the chromatin acetylation facilitates the recruitment of multiple coactivator complexes to gene promoters [44]. It has been repeatedly demonstrated that histone acetylation is connected with preinitiation complex formation [37]. In particular, acetylated histone H4 modifications, similar to H3K4me3, stabilize the interaction of general transcriptional factor TFIID with the promoter, since the double bromodomain module of the TAF1 subunit of this complex efficiently binds to the acetylated H4 tail [45]. Acetylation of histone H3 at Lys9 (H3K9Ac) and Lys14 (H3K14Ac) is also important for TFIID binding, with acetylation at Lys9 supporting the transition of the transcriptional complex to the elongation stage [46, 47].

Numerous data confirm the association of histone H3 trimethylation at Lys36 (H3K36me3) with transition from transcription initiation to productive elongation by Pol II [48]. This modification is correlated with the phosphorylation of the large Pol II subunit at Ser2 of its CTD repeat [49]. The productive elongation stage is also marked by histone H3 phosphorylation at Ser10 (H3S10P). Cell depletion of JIL-1 kinase, which is responsible for this modification, has been shown to interfere with Pol II transition to productive elongation [50].

As mentioned above, transcription initiation involves a dynamic circuit of chromatin modifications, with different covalent modifications of histones replacing each other. Another illustrative example is the cross-talk between histone H2B ubiquitination at Lys120 (H2BK120Ub) and histone H3 trimethylation at Lys4 (H3K4me3) in the promoter chromatin. It has been shown that H2BK120Ub is spread along the coding region of the actively transcribed genes, while H3K4me3 is locally concentrated in the distal promoter region and is dependent on histone H2B ubiquitination [40, 51]. Both these modifications are necessary for productive transcription elongation and appear to be introduced at least twice per transcription cycle [41, 52, 53]. This suggestion is based on fact that the process of transcriptional activation is dependent not only on enzymes that introduce these modifications but also on enzymes that remove them. For example, the SAGA deubiquitination module is important for the removal of repressive complexes and nucleosome destabilization on gene promoters, which is prerequisite to the earliest transcription initiation stages [54]. Thus, H2BK120Ub at the beginning of the transcription cycle plays a repressive role and must be removed to allow preinitiation complex formation, but this modification is introduced again at the late stages of the cycle to stimulate the process of transcription elongation [51, 53, 55]. As found recently, the dKDM5/LID demethylase, which specifically removes methyl moieties from H3K4me3, supports active transcription as does the deubiquitination module [56]. It may well be that histone H3 trimethylation at Lys4 also occurs twice per transcription activation cycle, initially contributing to the removal of repressive complexes and then, at the end, stimulating transcription elongation. The removal of both H2BK120Ub and H3K4me3 at the early stage of transcription initiation is functionally important, because disruption of this process results in reduction of Pol II phosphorylation at Ser5 [56]. This modification of the large Pol II subunit plays a pivotal role in mRNP complex formation, particularly in 7-methylguanosine (7-MeG) cap attachment at the 5′ end of the transcript [57].

Data regarding the correlation of deposition of covalent histone modifications with individual stages of the transcriptional activation cycle are summarized in Table 1. We assumed that the obtained information could be used to identify specific gene transcription stages occurring at a given moment. Recruitment of chromatin modifying coactivator complexes responsible for deposition of histone modifications is not discussed in detail but is summarized in Fig. 1 and Table 2 together with the coactivator complexes described below.

**Table 1:** Deposition of specific covalent histone modifications at particular stages of the transcription activation cycle

Type of modification	Stage of transcription cycle	Real or predicted effect on transcription activation	References
Methylation			
H3K4Me3	Repressor removal	Stabilization of NURF complex at the promoter	[9]
	GTFs recruitment	Stabilization of TFIID, enhances PIC formation and promoter clearance	[19–21]
	Productive elongation	Promotion of elongation at ATX1/AtCOMPASS–like regulated genes	[22, 32]
H3R17Me2	Activator recruitment	Association of TBP and TFIIA, definition of transcriptionally engaged promoter	[15, 16]
H3K36Me3	Productive elongation	Maintenance of hyper-phosphorylated Pol II, hypo-acetylation of the coding region	[31, 32]
		Suppression of histone exchange, regulation of alternative splicing	
H4R3Me2	Activator recruitment	Mobilization of the HATs and HMTs, definition of transcriptional competence	[15, 16]
Acetylation			
H3K9Ac	GTFs recruitment	Stabilization of TFIID	[20, 29, 30]
H3K14Ac	Activator recruitment	Mobilization of the HATs and HMTs; TFIID recruitment	[15, 20, 29]
H4R3Ac	Activator recruitment	Mobilization of the HATs and HMTs	[15]
H4K16Ac	GTFs recruitment	Stabilization of TFIID	[15]
Phosphorylation			
H1P	Repressor removal	Removal of histone H1 from the promoter	[9]
H3S10P	Repressor removal	Participation in primary rounds of chromatin remodeling	[9]
	Productive elongation	Facilitation of RNAP II release from promoter-proximal pausing	[33]
Ubiquitination			
H2BK120Ub	Transcription repression	Stabilization of promoter nucleosomes	[37]
	Productive elongation	Stimulation of the Pol II elongation	[34–36]

**Table 2:** Coactivator complexes functions at the particular stages of the transcription activation cycle

Family	Stage of action	Real or predicted effect during transcription activation stage	References
Promoter structure maintenance			
Mediator	Nucleosome removal	Stimulation of chromatin remodelers recruitment	[7, 49]
	GTFs recruitment	Stabilization of GTFs on promoter	[43]
	Pol II recruitment	Making GTFs competent for Pol II recruitment	[44]
Chromatin remodeling complexes			
SWI/SNF	Activator recruitment	Stimulation of activator binding	[15, 62, 63]
	Nucleosome removal	Promoter clearance from the nucleosomes	[9, 59, 60]
	GTFs recruitment	TFIID binding	[64, 65]
	RNA synthesis initiation	Pol II pausing	[66]
	Productive elongation	Modulation of transcription elongation and splicing	[15, 66–68]
ISWI	Activator recruitment	Promoter clearance from the nucleosomes	[9, 70]
CHD	Nucleosome removal	Stabilization of the open chromatin structure on promoter	[76–78]
	Productive elongation	Modulation of transcription elongation and splicing	[80, 82, 85]
Ino80	Nucleosome removal	Promoter clearance from the nucleosomes	[91, 92, 94, 95]
Histone-modifying complexes			
Methyltransferases			
Set1/MLL	Repressor removal	Trimethylation of Histone H3 on K4	[9]
	Productive elongation		[22, 32]
CARM1	Nucleosome removal	Mono and dimethylation of Histone H3 on R17	[15, 16]
PRMT1	Nucleosome removal	Mono and dimethylation of Histone H4 on R3	[15, 16]
Set2	Productive elongation	Trimethylation of Histone H3 on K36	[31, 32]
Demethylase			
KDM5/Lid	RNA synthesis initiation	Removal of H3K4me3 modification	[39]
Acetyltransferases			
SAGA	Activator recruitment	Acetylation of Histone H3 on K14	[15, 20, 29]
	GTFs recruitment	Acetylation of Histone H3 on K9 and Histone H4	[20, 29, 30]
CBP/p300	GTFs recruitment	Acetylation of Histone H4 at multiple sites	[15]
Ubiquitin ligase			
Bre	Productive elongation	Ubiquitination of H2B at K120	[34–36]
Deubiquitinase			
DUB module in SAGA	Repressor removal	Deubiquitination of H2B at K120	[9]
	Productive elongation		[38]

## The Mediator complex organizes coactivator complexes and GTFs at the promoter

Mediator is the largest transcriptional coactivator complex described to date [58]. This complex consists of 33 subunits, including Med 1–31, cdk8, and cyclin C. It is considered that Mediator operates as a regulatory hub of numerous signaling pathways governing the cell life cycle and plays a key role in coordination of signals providing for progression of different transcriptional stages [59]. Only one of its subunits (cdk8) has enzymatic activity, suggesting that this large coactivator complex has mainly a structural role in the transcription cycle, providing a platform for the formation of the preinitiation complex [60]. It has been shown that Mediator is recruited to interact with general transcriptional factor TFIID and activate the TFIID complex, making it capable of preinitiation complex formation and RNA polymerase II recruitment [61]. However, data on the involvement of Mediator in different stages of transcription are still incomplete, and this issue needs a more thorough investigation.

As shown in many model systems, recruitment of the Mediator complex is dependent on transcriptional activators; i.e., Mediator interacts with the promoter immediately after activator binding but prior to preinitiation complex formation [62]. Its recruitment is accounted for by the interaction between the tail module of this complex and specific activators, but this mechanism is not universal [63]. The middle and head modules of Mediator are responsible for its recruitment at the constitutively expressed genes [64]. The interaction of Mediator with the promoter does not depend on chromatin remodeling factors [65]. Conversely, recruitment of the SWI/SNF chromatin remodeling complex in yeast has proved to be dependent on the presence of mediator subunits [7, 66].

Thus, multiple data obtained to date make it possible to identify the exact transcriptional stage at which Mediator is recruited. Its binding to the promoter takes place immediately after activator recruitment but before the removal of promoter-bound nucleosomes. Therefore, Mediator functions as a transcriptional coactivator that is important for the recruitment of chromatin remodeling complexes and preinitiation complex formation, i.e., for the progression of the most important steps of transcription initiation.

The Mediator complex undergoes serious structural rearrangements in the course of transcriptional activation. It has been shown that the complete form of the Mediator complex, which includes the CDK8 module, interacts with the SAGA histone modifying complex but has no effect on the transcriptional process in vitro (i.e., is inactive). Whereas its truncated form which lacks the CDK8 module but contains an additional Med26 subunit is capable of stimulating in vitro transcription [67]. The CDK8 module was previously considered to be not involved in preinitiation complex formation, which is a major function of Mediator, the more so that subunits of this module were shown to have a repressive effect on transcription initiation [68]. According to recent data, however, CDK8 contributes to a novel function of Mediator, which is to support RNA polymerase II complex transition into the productive elongation phase [69].

The presence of the Mediator complex on the promoters of yeast genes is negatively correlated with chromatin acetylation at Lys16 of histone H4 (H4K16Ac) [70]. As noted above, this modification is introduced into the promoter chromatin at the stage of preinitiation complex formation and is important for its stabilization [18]. It may well be that H4K16Ac contributes to Mediator dissociation from promoters that contain an already formed preinitiation complex, where Mediator has accomplished its major task.

Recent studies provide evidence that an important role in the removal of Mediator complex from the promoter is played by CDK7/Kin28 kinase, which is also responsible for phosphorylation at Ser5 of the Pol II C-terminal domain [71]. CDK7/Kin28 kinase malfunction leads to considerable increase in the level of promoter-bound Mediator. Because of impaired dissociation of Mediator from the preinitiation complex, Pol II cannot make the transition from the initiation to elongation phase and leave the promoter region. As a result, the Pol II elongation rate decreases significantly.

The above data on the Mediator complex point out the importance of timely removal of coactivators in the course of the transcription cycle. We have previously noted that failure to remove chromatin modifications (the example of dKDM5/LID demethylase acting on H3K4me3) can also interfere with the transition into the elongation stage [56]. To all appearance, phosphorylation at Ser5 of the Pol II large subunit is one of major checkpoints of the transcription cycle. Coactivators and chromatin modifications that previously supported transcription initiation lose their significance at this stage and may even interfere with the onset of transcription elongation.

## Chromatin remodeling coactivators

The removal of nucleosomes from the promoter is one of the key stages of the transcription activation cycle. This stage is indispensable for the correct formation of the preinitiation complex and subsequent recruitment of Pol II. A number of coactivator complexes capable of chromatin structure remodeling have been described to date and, based on type of enzymatic activity of their ATPase subunits, classified into four families: SWI/SNF, ISWI, CHD, and INO80. Chromatin remodeling complexes have previously been regarded as frontline coactivators that are recruited at the earliest stages of transcription cycle and are important for clearing the promoter from heterochromatin and recruiting other coactivators. The results of recent studies show, however, that these complexes also participate in later stages of the transcription cycle, including transcription elongation. This is evidence for the insufficiency of our knowledge about chromatin remodeling coactivators. Apparently, they have additional, as of yet unknown functional characteristics that are realized after the basic function of a given coactivator is accomplished.

### SWI/SNF chromatin remodeling complex

SWI/SNF is a special type of chromatin remodeler that can affect nucleosomes in all known ways, causing their removal, sliding, loss of H2A/H2B dimers, and disruption of nucleosome interaction with DNA [72]. The SWI/SNF complex in eukaryotes is represented by two subclasses differing in several specific subunits that are important for the recruitment of the complex to chromatin, while most subunits are common to both subclasses and compose the core of the complex [73]. They include the enzymatic ATPase subunit SWI2 (in yeast) or its homolog Brahma (in *Drosophila* and humans). Mammals also have an additional ATPase subunit, named Brg1, which is structurally very close to Brahma but significantly differs from it functionally [74].

DNA-binding transcriptional activators are considered to play the leading role in SWI/SNF recruitment during promoter activation [75]. Several subunits of this complex contain protein domains that appear to be responsible for interactions with nuclear receptors and other transcriptional activators. Indeed, experiments in vitro and in vivo have confirmed that SWI/SNF interacts with activators of different types and is a very important component of different signaling pathways: STAT, hormonal (ecdysone, estrogen, progesterone, etc.), heat shock, and others [9, 18, 76–78]. Moreover, the SWI/SNF complex has an effect on the recruitment of transcriptional activators to the promoter [79, 80]. On this basis, we cautiously suggest that SWI/SNF is indeed recruited by the use of transcriptional activators but leave the possibility that the activator and coactivator also can interact beforehand and be recruited simultaneously, as a preformed complex. This recruitment hypothesis is supported by the results of detailed research on the transcription activation cycle. They show that the SWI/SNF chromatin remodeler is one of the first transcriptional coactivators to be recruited to the promoter and interact with it at the same time as transcriptional activator [18]. Moreover, SWI/SNF is recruited once again at the final stage of the transcription cycle. Its function at this stage is as yet unclear, but the authors of [18] suggest that SWI/SNF complex stimulates the nucleosome positioning process, which results in the closed structure of the promoter chromatin. Thus, the SWI/SNF complex appears to be one of the central factors in the transcription activation cycle that initially removes nucleosomes from the promoter, thereby activating the cascade of transcriptional processes, and then terminates this cascade by returning the promoter chromatin to the closed state. The exact mechanism of this termination is an interesting issue for further investigation. It may well be that the nucleosome positioned at the promoter site by the SWI/SNF complex is a functional molecular machine and the closure of chromatin structure initiate Pol II escape from the promoter and its transition to productive elongation.

Several studies have shown that the SWI/SNF remodeler and the transcriptional activator are recruited to the promoter simultaneously, as a preformed stable complex, and that this complex can also interact with other transcriptional coactivators [9, 18]. In particular, its interaction with TFIID results in the formation of a stable supercomplex, with both TFIID and SWI/SNF being recruited to the promoter in a mutually dependent way and functioning coordinately [81, 82]. Functional interdependence has also been demonstrated for SWI/SNF and Mediator coactivators [66]. Thus, the functioning of the SWI/SNF complex during the transcription cycle appears to be tightly connected with factors generating the preinitiation complex. The SWI/SNF coactivator apparently has other partners in the transcription machinery, but further investigations are needed to reveal them.

The exact mechanism of SWI/SNF removal from the promoter sequence is still unclear. Recent studies have provided data on the involvement of the SWI/SNF complex in the Pol II elongation process and on its physical association with the nascent pre-mRNP [83–85]. However, the question remains open whether the SWI/SNF complex involved in elongation is newly recruited to the activated gene or this is the same complex that stimulates the initiation process. Subsequent research will hopefully shed more light on the mechanism of SWI/SNF transition from the initiation to the elongation stage and its functional role in the elongation process.

### ISWI family of chromatin remodeling complexes

A distinctive feature of the ISWI coactivator family is a high diversity of complexes formed by the ATPase enzymatic subunit (named ISWI) shared by all members of this family. ISWI contains the catalytic DEXD ATPase domain together with two DNA-binding domains (HAND and SLIDE) and the histone-binding SANT domain and can interact with different proteins, forming various multiprotein complexes. The sets of such complexes differ between species. The family of the ISWI complexes described for humans is the most diverse and includes the ACF1, CHRAC, RSF, CERF, NoRC, WICH, WCRF, and NURF complexes [86]. It has been shown that most of them (specifically, ACF1, CHRAC, RCF, and WICH) participate in chromatin remodeling during replication and repair, while NoRC is involved in transcription repression. Here, attention will be focused on NURF, the complex of the ISWI family that has been shown to contribute to transcriptional activation of Pol II-dependent genes.

Although ample data point to the high biological significance of the NURF complex, the molecular mechanism of its action during transcriptional activation has not been studied in detail [87]. There is evidence that NURF can interact with nuclear receptors and other transcriptional activators. In particular, during transcriptional activation of progesterone-dependent genes, a stable complex consisting of NURF and nuclear receptors is considered to be responsible for promoter remodeling, which results in its clearing of heterochromatin and repressive complexes [9]. In certain genes, NURF appears to functionally substitute for the SWI/SNF coactivator and stabilize the interaction between transcriptional activator and promoter sequence. This remodeler forms a stable complex with the ecdysone receptor, and it has been found that mutations of NURF subunits mutations lead to malfunction of the *Drosophila* ecdysone cascade [88, 89]. These results characterize NURF as an important coactivator of ecdysone-dependent genes. A series of the genetic experiments have shown that NURF has a role in the Wingless (Wg) signaling pathway: it interacts with the Armadillo transcriptional activator to promote Wg-mediated transcription [90].

The role of histone modifications in the recruitment of chromatin remodelers has been best demonstrated for the NURF complex. It has been found that the BPTF/NURF301 subunit of NURF contains a PHD finger domain that binds to histone H3 trimethylated at Lys4 (H3K4me3) and that a decrease in the level of this modification results in the impairment of BPTF/NURF301 recruitment and consequent reduction in the level of chromatin-bound ISWI ATPase [91]. The results of kinetic research on transcriptional activation of progesterone-dependent genes activation confirm this finding: the NURF complex proved to be recruited to the promoters of these genes within a minute after their activation and removed on the second minute, with these events being in high correlation with the appearance and removal of the H3K4me3 mark in the promoter chromatin [9].

Thus, both the interaction of NURF with the activator and its binding to modified histone H3K4me3 may account for the recruitment of this complex, and further studies are needed to find out which of these factors plays the leading role.

### CHD family of the chromatin remodeling complexes

In coactivators of the CHD family, the enzymatic ATPase subunit contains the DEXD domain, as in other chromatin remodelers, but its distinctive feature is the presence of two additional chomodomains. In contrast to the families described above, the CHD family comprises complexes in which ATPase subunits slightly differ structurally [92]. On this basis, they are distinguished into three subfamilies: CHD1, with a C-terminal DNA-binding domain in the ATPase subunit; CHD3–5 (also called Mi2), without the DNA-binding domain but with two additional PHD fingers; and CHD6–9, a more diverse group of complexes containing SANT or BRK domains in the ATPase sequence [93].

The CHD1 complexes are the only group in the CHD family that has been shown to participate in transcriptional activation processes that take place at the promoters. CHD1 homologs in yeast have been found at the promoters of actively transcribed genes and supposed to be important for the stabilization of the open chromatin structure [94]. Mutations in the CHD1 enzymatic subunit have proved to cause a decrease in the transcriptional level of regulated genes [95, 96]. It is suggested that CHD1 complexes may be involved in the process of initial nucleosome disassembly during transcriptional activation, but the molecular mechanisms of CHD1 recruitment and participation at the early stage of transcription are unknown [93]. The functions of CHD1 complexes are not limited to transcription initiation at the promoters [97]. These coactivators are also involved in the Pol II elongation process: the CHD1 ATPase is a component of the elongation complex (along with subunits of the FACT and PAF complexes) and of the splicing machinery [98]. The interaction with PAF appears to be a leading factor in CHD1 recruitment at the transcription elongation stage [99]. The PAF complex is one of the latest participants of the transcription activation cycle and is recruited immediately before Pol II transition into active elongation. Therefore, CHD1 recruitment during the initiation stage is apparently independent of PAF and has a different, as yet unknown mechanism.

Complexes of the CHD3-5 (Mi2) subfamily contain specific subunits that have deacetylase activity, and their main function is considered to be related to transcriptional repression [100]. Indeed, Mi2 complexes are able to interact with the methylated DNA and participate in the cascade of events resulting in gene repression [101]. However, recent studies provide evidence for a novel function of the Mi2 subfamily. It has been shown that *Drosophila* Mi2 ATPase is recruited to the coding regions of actively transcribed heat-shock-dependent genes and is important for enhancing their transcription to the full extent [102]. The recruitment of Mi2 complex in this case is due to its interaction with poly (ADP-ribose), i.e., its mechanism is different from that in the repression cascade (where it is mediated by DNA-binding transcription factors). Unfortunately, the exact subunit composition of this complex and its functions during Pol II elongation are as of yet unclear.

According to available data, complexes of the CHD6-9 subfamily are not directly involved in the processes of gene activation at the promoters but specifically associate with transcriptional enhancers [103, 104]. A major role in this association is apparently played by chromodomains, which bind methylated histones concentrating in the chromatin surrounding the enhancers [104]. A Kismet/CHD8 ATPase complex has been found to participate in the Pol II elongation process, with *Kismet* gene mutation dramatically reducing the level of Pol II at the gene bodies while not affecting the level of its promoter-bound fraction [105].

Molecular mechanisms that control the recruitment of CHD complexes to the actively transcribed genes have not been studied adequately. In cases of Mi2 and CHD1, which participate in Pol II elongation, this process is mediated by PARP and PAF complexes, respectively, but the nature of their interaction with the promoter region during transcriptional activation is still obscure.

### Chromatin remodeling complexes of INO80 family

The INO80 family is composed of multiprotein complexes formed by SNF-2-like ATPases INO80 and SWR1 [106]. A distinctive feature of these complexes is that their enzymatic subunits are split into two parts. Another feature is that they contain DNA-helicase subunits (Rvb1 and Rvb2), actin, and actin-like proteins [107].

The results of in vitro experiments show that the INO80 complex causes nucleosome sliding and arrangement into a specific pattern, different from those obtained with other chromatin remodelers, and facilitates transcription, increasing its level up to tenfold [108, 109]. However, its function as a transcriptional coactivator in living cells is not so apparent. INO80 mutations in yeast result in repression of certain genes but activate genes of another group [110]. More detailed data on the mechanism of INO80 participation in transcriptional activation are available for only a few genes, in particular, *PHO5* and *INO1*. In the former, INO80 is recruited to the promoter region immediately after transcription induction, in a complex with SWI/SNF chromatin remodeler and SAGA histone modifier. These coactivator complexes are probably recruited to the chromatin via interaction with the corresponding DNA-binding transcriptional activator. The INO80 complex assists the SWI/SNF remodeler in clearing the promoter of nucleosomes and is important for complete transcriptional activation of *PHO5* [111]. The involvement of INO80 in transcriptional activation has also been demonstrated for the *INO1* gene, where its recruitment to the promoter chromatin is also mediated by a DNA-binding activator [112]. Evidence for the role of INO80 complexes in transcriptional activation is so far limited to these few facts and does not allow any conclusions about molecular mechanisms of their functioning. Further investigations will hopefully help to clarify this issue and find out at which stages of transcription INO80 is recruited and removed.

A major function of the SWR1 complex is to substitute the H2A/H2B dimer by a dimer containing histone H2A variants H2AZ [107]. Enrichment with this histone modification is characteristic of chromatin around the promoter regions of genes. It is considered that the variant histone H2A serves to form a barrier that separates the transcription initiation site from adjacent nucleosomes and is important for nucleosome positioning in the coding region of the gene [113]. Since there is no reliable evidence concerning the involvement of histone H2AZ in transcriptional activation, the SWR1 complex responsible for its substitution by the above variants cannot be yet regarded as a clear transcriptional coactivator.

## Conclusions

An analysis of available information on the involvement of coactivator complexes in individual stages of transcriptional activation has allowed us to propose a scheme illustrating the functions of coactivators at different stages of this process. Although consideration has been given to multiple data obtained with different gene models and organisms, we have revealed a substantial lack of information regarding recruitment/removal mechanisms for most of the analyzed complexes. The recruitment methods during the transcriptional activation cycle have been well described for some coactivator complexes. Transcriptional activators (in particular, nuclear receptors) are responsible for the binding of the SWI/SNF and NURF complexes, which mediate promoter clearance of occupied nucleosomes. Recruitment of the Mediator complex, a key structure organizing cross-talk between coactivators and general transcriptional factors, also occurs with participation of transcriptional activators. The involvement of the Mi-2 chromatin remodeler in transcription at active genes is mediated by the activity of PARP. Nevertheless, evidence for the participation of some coactivator complexes (such as CHD1, SWR1 and INO80) at the particular stages of this cycle is based only on scarce experimental data or even indirect evidence. The molecular mechanisms responsible for the removal of certain coactivator complexes after the termination of their functions at the promoters also remain unclear. Altogether, the proposed scheme of the transcription activation cycle can give an idea about the current state of knowledge in this field of molecular biology and point out the least studied issues for future research on coactivator complexes and the molecular mechanisms of their functioning. We believe that future investigation of the stages in which coactivators are recruited and removed will allow for significant improvement of integrated scheme of transcription activation process.

Kinetic studies of transcriptional activation will help to determine additional participants in the process. Even analogous molecular functions could be realized during gene activation by different coactivator complexes (just as the chromatin remodeling process at the promoter has been shown to be executed by different remodeler families). That is why the action of the coactivator complex recruited at the latter stages of the cycle is frequently hidden by the previous player with properties similar to those of the initial coactivator. Research on coactivator dynamics could uncover an answer to one of the current mysteries in the field: the accumulation of different transcriptional complexes in the same genomic regions. The temporal separation of the molecular functions of these complexes would help to determine the functional scheme of the particular genome region.

Recent advances in the visualization of the individual transcriptional processes in living cells have brought much clarity to the field. The real timing of transcription and the duration of its individual stages have become much clearer [25, 114]. Unfortunately, few inducible model genes have been studied with this modern single-cell technique at this moment [115, 116]. Some of the model genes that have been investigated have demonstrated a quick response immediately after the influence of the induction signal. It could be an extremely interesting task to reveal the molecular mechanisms that are occurring on the promoter and are responsible for the preparation of the gene for forthcoming activity. Based on the summarized data, the coactivator complexes that participate in transcriptional activation can stimulate different stages of the process. Thus, the transcriptional activation process could be represented as a sequence of the individual acts of the stimulation of the different stages of transcription. The exact order of these acts of stimulation and their molecular participants are currently completely undefined. Thus far, we can only hope that the rapid development of methods for investigations of individual genes in single cells will lead to the ability to explore the actions of coactivator complexes in the living process of transcriptional activation in the near future.
